# Mechanisms Involved in the Pro-Apoptotic Effect of Melatonin in Cancer Cells

**DOI:** 10.3390/ijms14046597

**Published:** 2013-03-25

**Authors:** Carmen Rodriguez, Vanesa Martín, Federico Herrera, Guillermo García-Santos, Jezabel Rodriguez-Blanco, Sara Casado-Zapico, Ana María Sánchez-Sánchez, Santos Suárez, Noelia Puente-Moncada, María José Anítua, Isaac Antolín

**Affiliations:** 1 Department of Morphology and Cell Biology, Faculty of Medicine, University of Oviedo, c/Julian Claveria 6, 33006 Oviedo, Spain; E-Mails: martinvanesa@uniovi.es (V.M.); fherrera@fm.ul.pt (F.H.); garciasantosph@gmail.com (G.G.-S.); JBRodriguez@med.miami.edu (J.R.-B.); saicz@hotmail.com (S.C.-Z.); sanchezmaria@uniovi.es (A.M.S.-S.); ssuarez@uniovi.es (S.S.); puentenoelia@uniovi.es (N.P.-M.); mjanitua@uniovi.es (M.J.A.); iantolin@uniovi.es (I.A.); 2 Oncology Institute of Asturias, University of Oviedo, 33006 Oviedo, Spain; 3 Institute of Molecular Medicine, Faculty of Medicine, University of Lisboa, Professor Egas Moniz Avenue, 1649-028 Lisboa, Portugal

**Keywords:** melatonin, cancer cells, apoptosis, ROS

## Abstract

It is well established that melatonin exerts antitumoral effects in many cancer types, mostly decreasing cell proliferation at low concentrations. On the other hand, induction of apoptosis by melatonin has been described in the last few years in some particular cancer types. The cytotoxic effect occurs after its administration at high concentrations, and the molecular pathways involved have been only partially determined. Moreover, a synergistic effect has been found in several cancer types when it is administered in combination with chemotherapeutic agents. In the present review, we will summarize published work on the pro-apoptotic effect of melatonin in cancer cells and the reported mechanisms involved in such action. We will also construct a hypothesis on how different cell signaling pathways may relate each other on account for such effect.

## 1. Introduction

The natural indoleamine, melatonin, is known to regulate light/darkness responses in living organisms from all kingdoms. However, the therapeutic potential of melatonin goes beyond the treatment of jet lag or sleep disorders; this indole showing neuroprotective and antitumoral properties in many experimental conditions [1–6].

The antitumoral effects of melatonin are observed at many different levels. The alteration of melatonin circadian rhythm in professionals doing night shifts increases the incidence of some kinds of tumors [7,8]. Conversely, melatonin concentrations in plasma are affected in cancer patients [9]. In experimental models of cancer, plasma concentrations of melatonin show oncostatic effects on tumors from a wide range of origins, by inhibiting tumor cell proliferation. Breast cancer is probably one of the most studied melatonin-sensitive tumors, and there are currently consolidated hypothesis explaining the intracellular signaling pathways involved in melatonin inhibition of breast cancer cell growth at plasma concentrations [10–12]. These data emphasize the possible importance of endogenous melatonin as an antitumoral agent.

Melatonin antitumoral effects in experimental models of cancer have been mostly studied using concentrations of the indole that are similar to those found in plasma, within the nanomolar range. Plasma concentrations are usually referred to as “physiological”. However, higher concentrations of this indole have also shown interesting effects in multiple diseases and conditions, including cancer. Said concentrations range from micromolar to millimolar, and authors frequently refer to them as “pharmacological” concentrations, since they are higher than plasma concentrations. However, in our opinion, the terms “physiological” and “pharmacological” should be revised. The term “pharmacological” has somewhat acquired negative connotations. Reviewers often argue that these concentrations are not physiological and that their use for human therapies is unrealistic. Melatonin concentration in the extracellular space remains unknown, and therefore, micro- or milli-molar concentrations of melatonin in the cell culture media may well be physiological. Additionally, saliva, bile and cerebrospinal fluid show higher melatonin concentrations than plasma, indicating a compartmentalization of the hormone that could result in its accumulation at micro- or milli-molar concentrations in cells and tissues [13,14]. For these reasons, and until these questions are addressed, we believe that the term “high” is more accurate to refer to micro/millimolar levels of melatonin and has less negative connotations than the term “pharmacological”. We will use this nomenclature throughout the present review.

Some cancer cells, such as androgen-independent prostate cancer cells or glioma cells, are insensitive to nanomolar concentrations of melatonin. However, their growth is inhibited by millimolar concentrations of the indole [15,16]. These data indicate that the oncostatic effects of melatonin could be extended to more cancer types by simply increasing its concentration. Furthermore, we and others have reported that high concentrations of melatonin induce apoptosis in certain types of cancer, such as Ewing’s sarcoma and most of types of hematologic cancer. However, the cytotoxic effect of melatonin seems to occur only in specific types of tumors and is not as extended as its oncostatic action.

Melatonin’s cytotoxic effect in particular tumoral cells is counter-intuitive, since high concentrations of melatonin also protect normal cells against oxidative damage, preventing the modifications of DNA, lipids and proteins exposed to oxidants [17–19]. In fact, inhibition of cell proliferation by high melatonin concentrations is, even in cancer cells, strongly associated with the antioxidant properties of the indole [16,20]. However, we and others have observed that high concentrations of melatonin enhance the effect of chemotherapeutic drugs, both in cultured cancer cells [21–31] and *in vivo*[32,33], suggesting that melatonin can be, in fact, also deleterious for cancer cells in particular conditions.

While the oncostatic effects of melatonin at nanomolar concentrations have been extensively studied and the pathways involved are relatively well characterized, the mechanisms of action of high melatonin concentrations and their cytotoxic effects remain poorly understood. This review focuses on the antitumoral effects of melatonin at high concentrations, with special attention to its cytotoxic effects on particular types of tumoral cells.

## 2. Pro-Apoptotic Effects of High Concentrations of Melatonin

The antitumoral effects of melatonin can be classified as cytostatic or cytotoxic. While both low and high concentrations of melatonin are able to stop the proliferation of tumoral cells, the cytotoxicity is observed exclusively at high concentrations of the indole. The kind of effect and its potency, however, depends strongly on the type of cell and tumor.

We have mentioned above that breast cancer cells are sensitive to plasma concentrations of melatonin and respond by stopping their proliferation [1,34]. Some cancer cells have little or no sensitivity to low, nanomolar concentrations of melatonin, but respond to higher concentrations by decreasing their proliferation markedly. While millimolar concentrations of this indole inhibit the proliferation of colon cancer cells, lower concentrations have no effect [35]. Nanomolar melatonin decreases the proliferation of ovarian CHO cells slightly (12%). However, at 1 millimolar, melatonin induces a striking decrease in their proliferation (50%) without inducing cytotoxicity [36]. Similarly, LNCaP human androgen-dependent prostate cancer cells show decreased proliferation (60%) after six days of treatment with melatonin 1 mM [15]. High, but not low, concentrations of melatonin also induce a decrease in the growth of the androgen-insensitive PC3 human prostate cancer cells, although not as markedly as androgen-dependent cells [15]. C6 rat glioma and A172 human glioblastoma cells do not respond to low concentrations of melatonin, but also stop proliferating without losing viability in the presence of millimolar concentrations of melatonin [16,37].

**Figure 1:**
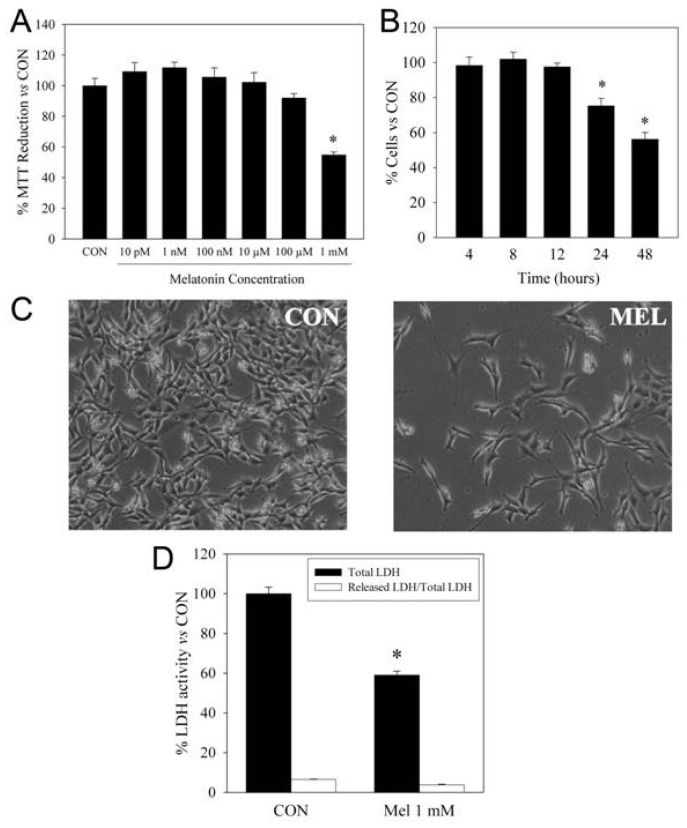
Melatonin effects on the hippocampal HT22 cell line. (**A**) Cells were incubated with several concentrations of melatonin. MTT reduction (which is proportional to the total number of cells) was determined after 24 h incubation. * *p* < 0.01 *vs.* all groups; (**B**) Cells were incubated with 1 mM melatonin for several time-points, and the MTT reduction was evaluated. * *p* < 0.01 *vs.* 4, 8 and 12 h incubation; (**C**) Cells were incubated with or without (control) 1 mM melatonin for 48 h and observed under phase contrast microscopy. The decrease of cell number without the increase of cellular debris is observed in cells treated with melatonin; (**D**) The decrease in total lactate dehydrogenase (LDH) (which is proportional to the total number of live cells) and no changes in the released LDH/total LDH ratio (which is proportional to the number of dead cells) was determined after 48 h of treatment with 1 mM melatonin.

Cytotoxicity is not observed in any of the examples shown above nor, to the best of our knowledge, in any type of non-tumoral cell. For example, melatonin (100 μM) inhibits neural stem cell proliferation and promotes their differentiation [38]. Human umbilical vein endothelial cells (HUVECs) also stop their proliferation in the presence of high melatonin concentrations [39]. Primary cultures of hepatocytes show growth arrest in the presence of high melatonin concentrations, but no cytotoxicity, even at concentrations as high as 10 mM [40]. HT22 is a non-tumoral mouse hippocampal cell line that also shows a marked decrease in its growth only at millimolar concentrations of melatonin (Figure 1). HT22 cell viability remains unchanged even at 2 mM melatonin, this being the maximum concentration tested in our laboratory, due to solubility issues.

**Figure 2:**
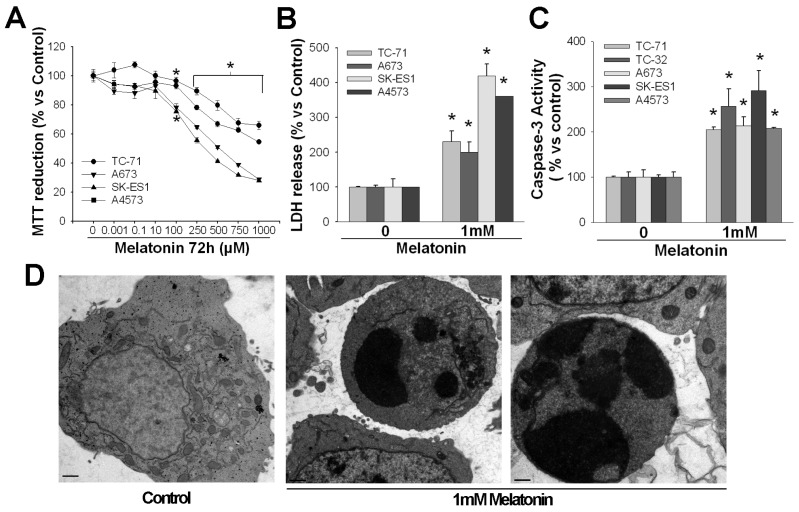
Melatonin effects on Ewing’s sarcoma cell lines. (**A**) Several Ewing’s sarcoma cell lines were incubated with increasing concentrations of melatonin. MTT (which is proportional to the total number of cells) was determined after 72 h. Melatonin concentrations over 100 μM decreased MTT. *p < 0.05 *vs.* vehicle treated groups; (**B**) Total LDH (which is proportional to the total number of live cells) and the released LDH/total LDH ratio (which is proportional to the number of dead cells) were determined after 72 h of incubation with 1 mM melatonin. All four cell lines studied showed an increase in cell death; (**C**) Caspase 3 activity was evaluated after 48 of incubation with 1 mM melatonin. It was increased in all four cell lines studied; (**D**) Electron microscope photographs of apoptosis induced by 1 mM melatonin in TC71 Ewing’s sarcoma after incubation for 72 h. The photograph on the left belongs to a non-treated TC71 cells. Bars: 1 μM.

**Figure 3:**
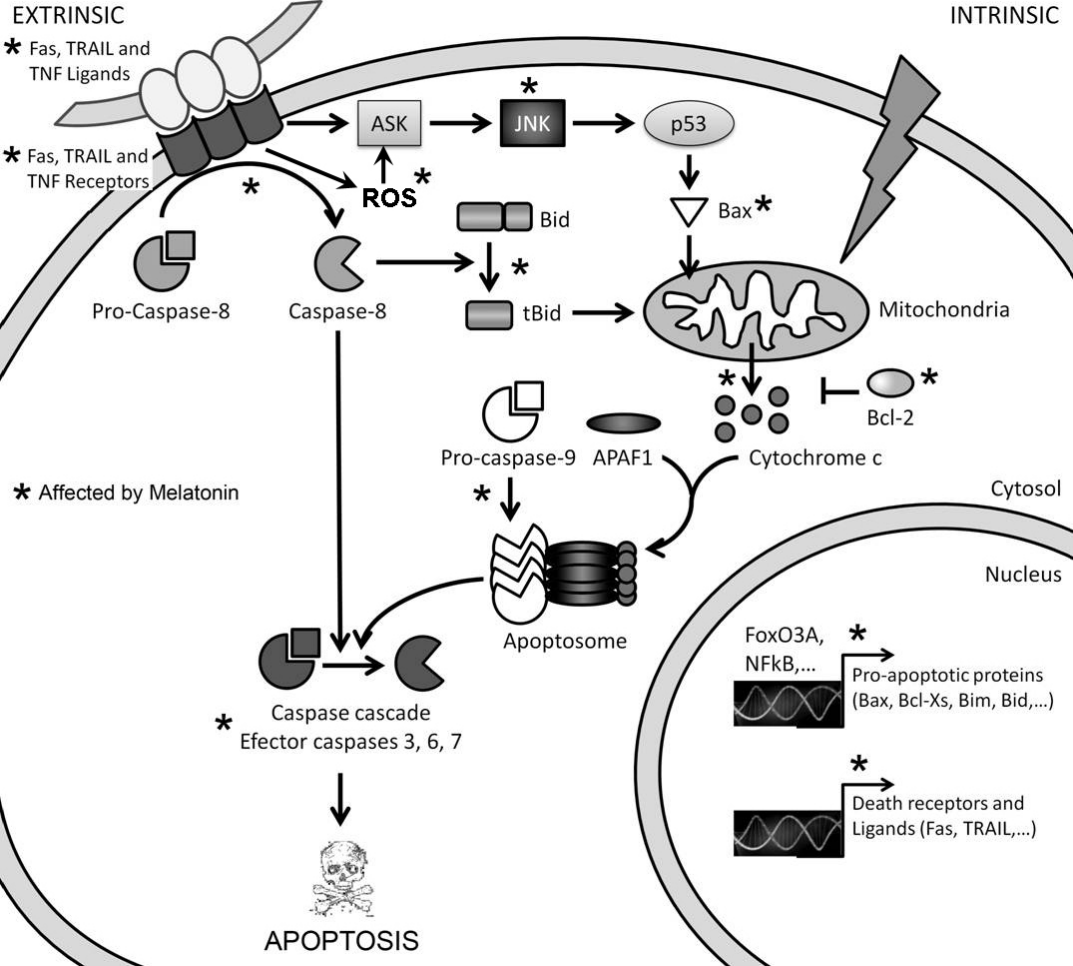
General view of the extrinsic and the intrinsic pathways of apoptosis. * Affected by melatonin.

A very restricted group of cancer cells show a cell death response after their treatment with high concentrations of melatonin, such cytotoxicity showing features corresponding to apoptosis (Figure 2). There are two main pathways to induce apoptosis, both of which seem to be involved, individually or in combination, in melatonin-induced cell death: the extrinsic or death receptor pathway and the intrinsic or mitochondrial pathway (Figure 3).

The extrinsic pathway is triggered by the binding of specific ligands from the TNF family (TNF, Fas ligand -Fas L-, TRAIL) to their receptors (death receptors: TNRF, Fas, Trail-R1, Trail-R2, DR3, DR6), which exist as trimers on the cell surface. The intracellular domain of death receptors (death domain), once activated by ligand binding, interacts with similar domains in particular adaptor molecules, bringing several of these molecules together and exposing their death effector domain (DED). The DED domain of adaptor molecules binds to the DED domain in caspase 8 monomers, resulting in dimmer formation and activation of this initiator caspase. The complex formed by the death receptor, the adaptor protein and the procaspase 8 is called the death-inducing signaling complex (DISC). Activated caspase 8 then cleaves executioner caspases (3, 6 and 7), which will act on their substrates, giving place to the morphological and biochemical features of apoptosis, or activates other proteins that allow crosstalk with the intrinsic pathway of apoptosis.

The intrinsic pathway of apoptosis relies on the disruption and permeabilization of the mitochondrial outer membrane (MOMP), which is followed by cytochrome c release and activation of the apoptosome. Activation by different stimuli of the pro-apoptotic members of the Bcl-2 family, such as Bax and Bak, are able to trigger MOMP. Such activation is controlled by other Bcl-2 family proteins (also pro-apoptotic), such as the activator BH3-only protein, Bim, or the de-repressor BH3-only protein, Bad. Anti-apoptotic proteins of the same family (Bcl-2, Bcl-XL and more) bind activator proteins in the absence of apoptotic stimuli, tightly regulating their activity and avoiding MOMP. Once MOMP occurs, cytochrome c is released into the cytosol and binds apoptotic protease activating factor 1 (APAF-1), which changes its conformation and exposes its oligomerization domain. Seven APAF-1 molecules bind to each other and form the apoptosome. This structure recruits procaspase-9 molecules and activates them, leading to the cleavage and subsequent activation of executioner caspases (-3, -6 and -7). As we mentioned above, the extrinsic pathway of apoptosis may engage the intrinsic one by caspase-8-mediated activation of proteins, such as the activator BH3-only protein Bid, which in turn activates Bax and Bak [41].

Hematological tumors are the best studied and the ones that present the most uniform results concerning the apoptotic effect by melatonin. Trubiani *et al.*[42] described the activation of caspase-3 after treatment with 2 mM melatonin in a cell line of Burkitt lymphoma (RAMOS). Such treatment also induced a decrease in the levels of the anti-apoptotic protein, Bcl-2, an increase in cytochrome c levels in the cytoplasm and an increase in mitochondria depolarization, all of them indicators of apoptotic cell death. Acute myeloid leukemia HL60 cells also show an increase in activated caspase-9 and caspase-3, the levels of the pro-apoptotic protein, Bax, and of cytochrome c released to the cytoplasm, as well as a decrease in Bcl-2 levels [43]. Similar results were observed later by Bejarano *et al.*[44,45] in acute myeloid leukemia (HL60) and chronic myeloid leukemia (K562) cells. The data from these three groups suggest that high concentrations of melatonin activate the intrinsic apoptotic pathway in hematologic cancer cells. However, they did not explore any parameter of the extrinsic pathway, and therefore, its involvement in melatonin’s cytotoxic effect cannot be ruled out.

We reported induction of apoptosis by 1 mM melatonin in acute myeloid leukemia (HL60), chronic myeloid leukemia (K562), Burkitt lymphoma (CA-46, RAMOS and RAJI) and acute lymphoid leukemia (RHE) cells [46]. Parameters from both the intrinsic and extrinsic apoptosis pathways were studied, showing an increase in the activation of caspases 3, 7 (executor caspases), 8 (initiator caspase of the extrinsic pathway) and 9 (initiator caspase of intrinsic pathway). It is noticeable that the increase in caspase-8 activity was much more pronounced than the increase in caspase-9 activity. The levels of activated proapoptotic protein, Bid, which is activated by caspase-8 and involved in the activation of the mitochondrial pathway of apoptosis, were also increased. Furthermore, the expression of both Fas and its ligand, FasL, limiting upstream factors of the extrinsic pathway, was highly increased after melatonin treatment. Recent studies on lymphoid cancer cells [47] showed activation of both caspases, 8 and 9, in two of the cell lines studied (RAMOS and SU-DHL-4), while only caspase 9 was activated in DoHH2 and JURKAT cells.

These data strongly support that both the intrinsic and extrinsic pathways of apoptosis are activated by millimolar concentrations of melatonin in hematological cancer cells. Interestingly, melatonin sensitizes human glioma cells to TNF-related apoptosis-inducing ligand (TRAIL)-induced cell death, cells that otherwise respond to melatonin by stopping their growth [26]. Further studies should be done to elucidate the interplay between these two apoptotic pathways in cancer cells upon melatonin treatment, since different pathways may be involved in the effect on different types of cancer or even on different cell lines.

A cytotoxic effect by millimolar concentrations of melatonin was also found in Ewing’s sarcoma cells, although not in other types of sarcoma [37,48]. Although we originally assumed that SK-N-MC cells were neuroblastoma cells, they are currently considered as Ewing sarcoma cells, based on the translocation that gives rise to the Ews-Fli1 protein and other molecular features. Later on [49], we further characterized this cytotoxic effect as apoptosis and associated it to the activation of the extrinsic pathway (*i.e.*, the increase in the expression of Fas/Fas L and the proapoptotic gene, Bcl-Xs, and caspase-8 activation). Results were confirmed in various Ewing sarcoma cell lines.

Other cancer types seem to die in the presence of high concentrations of melatonin, but the results are more controversial. One of the first reports on melatonin-induced cell death was by Winczyk *et al.*[50], who reported induction of apoptosis by melatonin in colon cancer *in vivo*. However, their results have not been confirmed by other laboratories, and colon cancer cells seem to respond to high melatonin concentrations mainly by reducing their proliferation [22,35].

Ozdemir *et al.*[51] showed inhibition of cell proliferation, but not apoptosis, after the treatment of HepG2 liver cancer cells with melatonin 1 mM. However, Martín-Renedo *et al.*[52] showed apoptosis induced by 1 mM of the indole in the same cell line. They found cell death accompanied by activation of caspases 8, 9 and 3; and an increase in the levels of the proapoptotic protein, Bax, and cytosolic cytochrome c. They did not analyze Fas/Fas L expression or other cell death receptors or ligands, but their results clearly involved the extrinsic apoptotic pathway. Recently, the same group showed that melatonin upregulates the proapoptotic protein, Bim, through the increase of FoxO3a transcriptional activity in the same cells [53].

Results on prostate cancer cells are also controversial. Joo and Yoo [54] showed induction of apoptosis by melatonin 1 mM in androgen-dependent LNCaP prostate cancer cells, as determined by the binding of annexin-V. Later on, the same group [55] showed activation of caspase 8 accompanied by an increase in the levels of Bax and a decrease in the levels of the anti-apoptotic protein, Bcl-2. However, these results contradict others previously reported by Sainz *et al.*[15] in the same cell line. Even though Sainz *et al.* also found an important MTT reduction in the same cell line after treatment with 1 mM melatonin, they found no decrease in cell viability by means of trypan blue assays, suggesting that such a decrease in cell number is due to a cytostatic and not a cytotoxic action of melatonin.

A decrease in cell number together with an increase in caspase 3 activity were found by Gonzalez *et al.*[56] in a rat pancreas cancer cell line (AR42J) treated with millimolar concentrations of melatonin. These results remain to be confirmed in other pancreatic cancer cells and by other groups, but, given the deadly nature of pancreatic cancer, we believe further studies should definitely be carried out in this direction.

Other tumoral cells show no response whatsoever to either low or high melatonin concentrations. For example, Sanchez-Sanchez *et al.*[37] found no changes in cell proliferation or viability in A549 human pulmonary adenocarcinoma cells. However, due to the general tendency to publish only positive results, it is not easy to find studies where non-sensitive cells are reported. While our experience is that most cells respond to at least high concentrations of melatonin, the number of non-sensitive cells could be greatly underrated, due to the lack of published evidence. Such cells could be extremely useful to define the molecular determinants of melatonin effects, which should obviously be present in sensitive cells and absent in non-sensitive cells.

In summary, melatonin-induced apoptosis only occurs at high concentrations of the indole and only in specific cancer types. Both the extrinsic and intrinsic pathways of apoptosis can be activated by melatonin, but little is still know about the exact contribution of each pathway to effects of the indole.

## 3. Mechanisms of Action Involved in the Pro-Apoptotic Effect of High Concentrations of Melatonin

One of the greater difficulties found in mechanistic studies at cellular and molecular levels are the constant intersection and crosstalk between signaling pathways, since most proteins are involved in several of them. It took years to explain the intracellular signaling pathways involved in the cytostatic effects of low concentrations of melatonin, which works mainly via its membrane receptors [12]. It is therefore not surprising that the mechanisms underlying the pro-apoptotic effects by high concentrations of melatonin—much higher than it is necessary to bind its receptors—remain to be unraveled after less than a decade of efforts.

### 3.1. Melatonin-Induced Apoptosis Is Associated with an Early Increase of Reactive Oxygen Species (ROS)

As melatonin has antioxidant properties at high concentrations, several reports on melatonin apoptotic effects studied the oxidative status of cells upon treatment with the indole. Opposite to what was expected, researchers found that there is an early increase in oxidative stress in those cancer cells, where melatonin induces apoptosis, besides the late increase of intracellular oxidants associated to apoptosis itself. Osseni *et al.*[57] reported that only 45 min after treatment with melatonin at concentrations higher than 100 μM, there was an increase in the production of reactive oxygen species (ROS) in HepG2 cells. This increase was concomitant to a loss of cell viability, although they did not analyze whether there was actually apoptosis. Wolfler *et al.*[58] also found an increment in ROS production in Jurkat cells after less than one hour of treatment with melatonin (500 μM–2.5 mM). Such an effect was prevented by antioxidants (*i.e.*, glutathione or trolox). The observed increase in ROS production induced by melatonin corresponded with a potentiation of later ROS increments and FasL-induced toxicity. However, they did not analyze the possible pro-apoptotic effect of melatonin by itself. Later on, Buyukavci *et al.*[59] found a correlation between the increase in ROS production and the induction of apoptosis by melatonin in several cell lines of hematological cancer. However, they used very early points to evaluate apoptosis, and we think their results would be even more pronounced if they used longer time points. Also, using cell lines of hematological cancer (AML HL60 cells and CML K562 cells), Bejarano *et al.*[45] found that the increase in ROS production two hours after treatment with 1 mM melatonin corresponded to the decrease in cell viability and the activation of caspases 9 and 3. All of these changes were prevented by antioxidants (*N*-acetylcysteine, trolox, catalase or glutathione). Casado-Zapico *et al.*[46] showed an increase in ROS production previous to various apoptosis-related events analyzed in AML (HL60), CML (K562) and Burkitt (CA46) cell lines treated with 1 mM melatonin. More specifically, we observed an increase in the levels of death receptors and their ligands and activation of caspases from both the extrinsic and the intrinsic pathways. We reported similar results [49] in many Ewing sarcoma cell lines, also showing that additional treatment with antioxidants prevented ROS production and the decrease in cell viability.

ROS form part of several signaling pathways activated by intra- or extra-cellular stimuli. At low levels, they can induce an increase in cell proliferation; while at high levels, they can induce apoptosis [60]. The role of ROS in apoptosis can follow different pathways, even in the same cell type [61]. First, ROS can induce apoptosis through the mitochondrial pathway, by interacting with proteins of the mitochondrial permeability transition complex [62]. Oxidative modification of such a complex can collapse the mitochondrial membrane potential and trigger the translocation of Bax and Bad pro-apoptotic proteins and the release of cytochrome c to the cytosol [63]. This is consistent with the observations by us and others in cancer cells that are sensitive to melatonin, particularly in liver cancer cells [52,57], acute myeloid leukemia cells [43,44,46] and Burkitt lymphoma cells [42,46].

**Figure 4:**
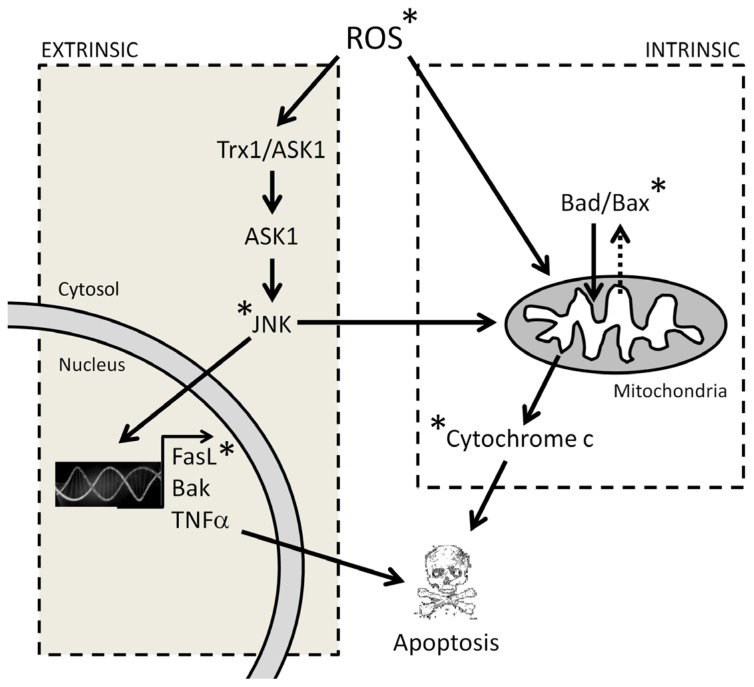
Roles of ROS in apoptosis. * Affected by melatonin.

ROS can also activate the ASK/JNK signaling pathway and drive cells to apoptosis. It has been previously proved that the Trx1/ASK1 complex works as a redox sensor. When Trx1 is reduced, it prevents ASK1 activation and the apoptotic cascade [64]. On the other hand, in an oxidized environment with high ROS levels, Trx1 splits from the complex and allows ASK1 activation. ASK1 then oligomerizes and promotes the formation of a complex with ASK2 and TRAFF2/6, which in turn activates JNK and p38 [65]. Translocation of activated JNK to the nucleus promotes the expression of pro-apoptotic proteins, such as TNF alpha, FasL or Bak, and, consequently, the extrinsic apoptotic pathway [66]. On the other hand, JNK translocation to the mitochondria promotes cytochrome c release and, consequently, the intrinsic apoptotic pathway [67]. Although this pathway still has not been analyzed systematically in all cell lines where high concentrations of melatonin induce apoptosis, there is evidence indicating that JNK and p38 activation could be involved in melatonin-induced apoptosis, at least in some cancer cell types. Martin Renedo *et al.*[52] found an increase in the activation of JNK 1, 2 and 3 and p38 after the treatment of liver cancer cells with 1 mM melatonin. The activation of these kinases was accompanied by an increase in the levels/activity of p53, p21 and caspases 8, 9 and 3, as well as by cytochrome c release. Joo and Yoo [54] observed JNK and p38 activation by 1 mM melatonin in LNCaP androgen-dependent prostate cancer cells. Inhibitors of these kinases prevented melatonin-induced cell death. Later on, they also demonstrated that the activation of these kinases was responsible for the increment in p21, p27 and p53 levels [55]. Both, leukemia and Ewing’s sarcoma cell lines show Fas L expression increase [46,49], which could be also related to the activation of this pathway by ROS, although JNK activation was not analyzed in these reports. A summary of the possible ROS-related pathways in the pro-apoptotic effect of melatonin is represented in Figure 4.

### 3.2. The Early Increase of ROS Production Determines if Cancer Cells Die or Stop Proliferating in Response to Melatonin

The data mentioned above suggest that the increase in ROS production is related to the pro-apoptotic effect of high concentrations of melatonin in cancer cells. We analyzed several parameters related to the intracellular redox state in a recent study [37], comparing cancer cell lines that die by apoptosis upon treatment with melatonin, cancer cells that decrease their proliferation in the same conditions and cancer cells that are not sensitive to melatonin. Intracellular levels of ROS increased early in cells that died by apoptosis, decreased after 48 h in cells that diminished their proliferation and did not change in non-sensitive cells. Results on intracellular glutathione (GSH) were consistent with ROS levels in all cell lines. Cells that die by apoptosis showed decreased GSH levels; cells that stop proliferating showed increased GSH levels; and non-sensitive cells did not present any change. Variations in GSH levels occurred sometime after ROS levels changed, indicating that such variations are not the cause of ROS production, but probably a consequence of it. Finally, cells undergoing apoptosis presented decreased expression of antioxidant enzyme expression, probably as a consequence of the increase in ROS production, although they recover after 48 h of treatment. The opposite occurred in cells that decrease their proliferation in response to melatonin, and no changes were found in non-sensitive cells. Administration of antioxidants prevented melatonin-induced apoptosis, while hydrogen peroxide enhanced it. It seems, therefore, that the induction of apoptosis by high concentrations of melatonin in sensitive cancer cells is related to the increase in ROS levels and, given the early appearance of such phenomenon, it is likely to be a key factor in melatonin-induced cell death.

### 3.3. The Reduction in Intracellular GSH Levels Could also Be Involved in the Pro-Apoptotic Effect of Melatonin

While melatonin increases GSH levels in many cell types, both normal and tumoral, it produces the opposite effect in cells that undergo apoptosis. The decrease in intracellular GSH levels could be due to its oxidation by ROS, but also to its escape out of the cell [68]. It has been found in other cell types that γ-glutamyl transferase catalyzes the hydrolysis of extracellular GSH and, thus, induces the release of intracellular GSH reservoirs [69]. FasL also induces the release of cellular GSH in lymphoid cells, which is essential for FasL-dependent apoptosis [68]. These authors also observe that the increase in ROS levels is a side effect, rather than an event necessary for apoptosis. A decrease in intracellular GSH levels could also be due to *de novo* reduction of its synthesis. An arrest in GSH synthesis induces a type of apoptosis dependent on activation of protein kinase C (PKC) [70], while a decrease in GSH levels due to its release to the extracellular medium induces JNK-dependent apoptosis [71]. Although the decrease in GSH levels seems to be secondary to elevated ROS production in the case of melatonin-induced apoptosis [37], the involvement of GSH in melatonin cytotoxic effects deserves deeper study. GSH depletion by high concentrations of melatonin was reported by Albertini *et al.*[72] in U937 human leukemia cells, although these authors did not study whether melatonin also induced cell death. Casado-Zapico *et al.*[46] found Akt activation (a protein kinase downstream PKC) to be involved in melatonin-induced apoptosis in hematological cancer cell lines. Interestingly, melatonin sensitizes human glioblastoma cells to TRAIL-induced cell death by a pathway involving PKC and Akt [26]. JNK activation by melatonin has been found in liver cancer cells and androgen-dependent prostate cancer cells [52,54]. However, JNK is sensitive to redox changes, and its activation could therefore be triggered by the increase in ROS production. In summary, we believe further studies should be carried out in order to elucidate the exact role of ROS, GSH and apoptotic pathways in melatonin-induced cancer cell death.

## 4. Conclusions

Current evidence indicates that alterations of the intracellular redox state play a key role in the effects of high concentrations of melatonin in cancer cells, reducing conditions being associated with a decrease in cell proliferation and oxidative conditions with apoptosis. The last data may be in conflict with the fact that high concentrations of melatonin show clear antioxidant properties. Furthermore, unlike other antioxidants, such as vitamins C or E, melatonin oxidation results in metabolites that still possess antioxidant properties [73–78]. Thus, the key question is: why are ROS elevated in some cancer cells upon their treatment with high concentrations of melatonin? We believe that a primary pro-oxidant effect of this indole could be ruled out, bearing in mind the data exposed above and the fact that this increase in ROS is not a general fact, but an exception. A fact to be considered is that the metabolism of cancer and normal cells shows numerous differences. Metabolism can also differ between different types of cancer. However, these are only speculations, and the actual explanation remains to be found. We believe that addressing this question is essential to understand the mechanisms involved in the antitumoral apoptotic effects of melatonin and that it will be a major challenge in the field for the next few years. The fruits of such efforts could contribute toward improving therapies for at least some particular types of cancer.
